# Effect of Feeding Sugarcane Bagasse-Extracted Polyphenolic Mixture on the Growth Performance, Meat Quality, and Oxidative and Inflammatory Status of Chronic Heat-Stressed Broiler Chickens

**DOI:** 10.3390/ani14233443

**Published:** 2024-11-28

**Authors:** Motoi Kikusato, Fu Namai, Katsushige Yamada

**Affiliations:** 1 Laboratory of Animal Nutrition, Graduate School of Agricultural Science, Tohoku University, Aramaki Aza-Aoba 468-1, Sendai 980-8572, Miyagi, Japan; 2 Laboratory of Animal Food Function, Graduate School of Agricultural Science, Tohoku University, Aramaki Aza-Aoba 468-1, Sendai 980-8572, Miyagi, Japan; fu.namai.a3@tohoku.ac.jp; 3 Advanced Materials Research Laboratories, Toray Industries, Inc., 10-1, Tebiro 6-chome, Kamakura 248-8555, Kanagawa, Japan

**Keywords:** gut health, intestinal permeability, inflammation, phytochemicals, heat stress, broiler chickens

## Abstract

Background: This study aimed to evaluate the effects of sugarcane bagasse-extracted polyphenolic mixture (SBPM) supplementation on the harmful effects of chronic heat stress (HS) in broiler chickens. Methods: Two hundred and eighty-eight day-old male Ross 308 chicks were fed an SBPM in 0, 75, 150, or 300 ppm-supplemented diets and reared under thermoneutral (TN, 22.1–24.8 °C) or chronic HS (28.3–36.2 °C) conditions from 11 d to 42 d. Results: The chronic HS treatment negatively affected body weight, feed intake, and feed conversion ratio (*p* < 0.05), and these changes were partially attenuated by the SBPM supplementation (*p* < 0.05). Plasma lipid peroxidation content, inflammatory cytokines [interleukin (IL)-6, IL-β], corticosterone, and uric acid concentrations were significantly increased by HS, and these increases were attenuated by the SBPM supplementation (*p* < 0.05). Intestinal permeability indicator and serum fluorescein isothiocyanate-dextran levels after oral gavage were increased by HS and were also suppressed by the supplementation (*p* < 0.05). The HS-decreased muscle drip loss, lipid peroxidation, and glutathione content were also suppressed by the SBPM supplementation. The abovementioned alleviating effects of the SBPM were of a dose-dependent manner in most cases. Conclusion: This study demonstrated that SBPM supplementation can improve the growth performance, meat quality, inflammation, and intestinal permeability of chronic HS-treated broiler chickens.

## Simple Summary

The management of heat stress (HS) in domestic animals has been an important issue for sustainable meat production in the era of accelerated global warming. Chickens are more susceptible to high temperatures than other livestock animals because they lack sweat glands and heat dissipation is limited to the face, legs, and comb, which are not covered with feathers. In addition, the lower ratio of body surface area to body weight in chickens, especially broilers, is negatively associated with body temperature control under HS conditions. The negative consequences of HS include reduced body weight gain, feed intake, higher feed conversion ratio, and loss of meat quality. Multiple functional additives have been used to enhance the ability to counteract HS. Increased oxidative stress and inflammatory responses are some of the symptoms observed in HS-treated chickens, and the additive for minimizing these negative consequences has been strongly required. The present study demonstrated that feeding sugarcane bagasse-extracted polyphenolic mixture supplementation improves the growth performance, inflammatory and oxidative statuses, intestinal permeability, and meat quality of broiler chickens exposed to chronic HS.

## 1. Introduction

Global poultry meat production has rapidly increased to meet the increased demand for animal proteins [1]. However, accelerated global warming has impacted the poultry sector and threatened sustainable production. High temperature is one of the significant stressors negatively influencing poultry production and health. Heat stress (HS) is caused when the core body temperature continuously exceeds the range of the thermoneutral zone, resulting in growth retardation and metabolic/immunity dysfunctions. Increased oxidative stress and inflammatory responses are symptoms observed in HS-treated chickens [2,3]; both of these could be associated with growth retardation and a loss of meat quality in broiler chickens [4]. Oxidative stress is due to the imbalance between free radical generation and antioxidant capacity. The inflammatory response is mainly caused by intestinal pathogens introduced into the circulation. Therefore, it is likely that the augmentation of antioxidant ability and inhibition of the pathogen invasion may play an important role in suppressing the negative effects of HS.

Functional additives, such as vitamins/minerals [5], polyphenols [6,7], polysaccharides [8], and amino acids [9], have been widely used to potentiate immunomodulating or antioxidative abilities to counteract the HS effects. Sugarcane bagasse (SB) is a dry, fibrous material obtained from sugarcane (*Saccharum officinarum* L.) after squeezing the juice for sugar production. The residue comprises 35–50% cellulose, 20–35% hemicellulose, and 10–25% lignin, with the values differing in the production area [10,11]. As lignin consists of phenolic crosslinked polymers, its extraction can yield a variety of (poly)phenolic compounds [12]. It has been reported that the administration of SB extract has exhibited therapeutic effects on the lesion score of the *Eimeria* challenge [13], cyclophosphamide-induced inflammation in chickens [14], and endotoxin shock in mice [15].

From these lines of evidence, it is hypothesized that SB-extracted polyphenolic mixture (SBPM) supplementation could alleviate the harmful effects of chronic heat stress (HS) in broiler chickens. The present study investigated the effects of incremental supplementation of the SBPM on the feed growth performance, meat quality, inflammatory status, and intestinal permeability of chronic HS-treated broiler chickens.

## 2. Materials and Methods

### 2.1. Preparation of the SBPM

Sugarcane bagasse was treated with an alkali solution (1% sodium hydroxide solution for 2 h), and the soluble fraction was adjusted to a pH of under 4.0 and precipitated by 35% hydrochloric acid. The insoluble fraction obtained was made dry and powdered. The SBPM consisted of 5–10% polyphenol, <5% moisture, 50–70% diatom earth, and other constituents. The oxygen radical absorbance capacity (ORAC) value was more than 10 μmoles per g as a Trolox equivalent.

### 2.2. Animals and Experimental Design

Two hundred and eighty-eight day-old male chicks (Ross 308, *Gallus gallus domesticus*) were obtained from a commercial hatchery (Matsumoto Poultry Farms & Hatcheries Co., Ltd.; Miyagi, Japan). Chicks with similar average body weights were randomly distributed into four treatment groups: basal diet (SBPM, 0 ppm) and diets supplemented with 75, 150, and 300 ppm of the SBPM (Cellulosic Biomass Technology Co., Ltd., Bangkok, Thailand). At 11 d, the chicks in each diet group were further divided into two groups, each of which was reared at thermoneutral [TN; 19.8–24.3 °C (average of 21.1 °C)/39.8–50.2 relative humidity (RH)%] or constant HS conditions [31.7–35.4 °C (average of 33.2 °C)/51.3–64.5 RH%] until 42 d. Each treatment group consisted of 6 replicates, with six birds per replicate reared on a 0.81 m^2^ (0.9 m × 0.9 m) floor. The birds were reared under a 23 h light: 1 h dark cycle and provided ad libitum access to water and feed. After the HS treatment began, the rectal temperature of a bird randomly chosen from a pen was routinely monitored twice a day (9:00 a.m. and 5:00 p.m.) using a needle thermometer. 

The diet compositions are shown in Table 1, and each nutritional level followed the breeder’s recommendation. All the diets were made in mash form. Body weight (BW) and feed intake (FI) were monitored at 10, 25, 35, and 42 d. At 42 days of age, two birds that exhibited similar BW to the average values of the pen were selected from each pen and euthanized by decapitation for the subsequent biochemical analyses. Blood and skeletal muscle tissues were collected and stored at −80 °C until analyzed. 

### 2.3. Determination of Blood Oxidative Stress, Inflammatory Parameters, and Intestinal Permeability

Blood was collected in heparinized tubes and centrifuged at 825× *g* for 15 min at 4 °C to isolate plasma. The plasma corticosterone (CORT), IL-6, IL-1β, and uric acid (UA) concentrations were measured using a commercial kit [#ADI-900-097 (CORT), Enzo Life Sciences, Farmingdale, NY, USA; #MBS2021018 (IL-6), MBS2024496 (IL-1β), MyBioSource, Inc., San Diego, CA, USA; #437-17301 (UA), Fujifilm Wako Pure Chemical Corporation, Osaka, Japan] according to the manufacturer’s instructions. Plasma 2-thiobarbituric acid reactive substance (TBARS) values were determined as a lipid peroxidation indicator as previously described [16]. 

Serum fluorescein isothiocyanate-dextran (FITC-d) levels after oral gavage are used to evaluate intestinal permeability. The permeability of the compound depends on the barrier dysfunction, with higher values indicating greater permeability. The FITC-d levels were assessed as previously described [17]. Briefly, chickens were fasted for 12 h before the oral administration of a FITC-d solution (#FD4; Sigma Aldrich Co., St. Louis, MO, USA; 2.2 mg/kg BW). After 2.5 h, blood was collected and placed for 3 h at 21–24 °C. Serum samples were collected by centrifugation at 1500× *g* for 15 min at 4 °C and diluted to 1:1 in phosphate-buffered saline. Serum FITC-d levels were measured at excitation and emission wavelengths of 485 nm and 528 nm, respectively, using a spectrofluorimeter (RF-5300PC; Shimadzu Co., Kyoto, Japan). Fluorescence intensity was determined from a standard curve with known FITC-d concentrations. 

### 2.4. Evaluation of Meat Quality 

Skeletal muscles, pectoralis major, and biceps femoris were immediately taken from the birds sacrificed. A portion of each muscle tissue, approximately 5 g and with a similar surface area, was hung with a wire in a plastic case and stored at 4 °C for 3 days. The exudate was carefully wiped after storage, and the weight loss was measured and divided by the initial weight to obtain a percentage of drip loss:

Drip loss (%) = [(initial weight − the weight after cold storage)/initial weight] × 100

The muscle oxidative damage was determined by measuring the TBARS values mentioned above [16]. The glutathione content in the skeletal muscles was also measured as one of the meat quality parameters by using a commercial kit (#342-09011; Fujifilm Wako Pure Chemical Corporation, Osaka, Japan) according to the manufacturer’s instructions.

### 2.5. Statistical Analysis

All data were analyzed using Bell Curve (Social Survey Research Information Co., Ltd., Tokyo, Japan). Data are presented as the means of six replicates (growth performance), twelve individual birds (blood parameters, gene expression, meat quality), or six birds (microbiota). The data were analyzed by using one-way (growth performance at the stater phase) or two-way (parameters excluding the above parameter) analysis of variance (ANOVA) with the Tukey multiple-comparison test. Pearson’s coefficient analysis was used to analyze the correlation in the meat quality parameters. Differences were considered significant for values with *p* < 0.05.

## 3. Results

### 3.1. Effects on Growth Performance and Body Temperature

The results of the growth performance are shown in Table 2. The SBPM supplementation did not affect the BW and body weight gain (BWG) at the stages under the TN conditions, except for the BW at 35 d (*p* > 0.05). Chronic HS significantly reduced the BW and BWG at any stage, while the SBPM supplementation attenuated the reduction. The SBPM supplementation did not affect the FI at the stages under the TN conditions, except at 26–35 d. The reduction in the FI due to the HS treatment was attenuated by the SBPM supplementation, except at 36–42 d (*p* < 0.05). The alleviating effects of the SBPM supplementation on the BW, BWG, and FI in the HS conditions were partially dose-dependent. The SBPM supplementation significantly increased the FCR values for 26–35 d; however, it did not affect the FCR values at the other stages (*p* > 0.05). For the whole period (0–42 d), the SBPM supplementation improved all the performance parameters under the HS conditions, though there were a few differences in the periods exhibiting the effects. 

The average body temperatures of the birds during TN and HS conditions/treatment were 41.3 ± 0.2, 41.2 ± 0.2, 41.4 ± 0.3, and 41.3 ± 0.4 °C (TN) and 43.2 ± 0.4, 42.9 ± 0.3, 42.8 ± 0.4, and 42.6 ± 0.5, with an order of groups supplementing SBPMs of 0, 75, 150, 300 ppm. The HS treatment significantly affected the rectal temperature; however, there were no significant effects of the SBPM supplementation on the temperature.

### 3.2. Blood Biochemical Parameters

This study measured plasma TBARS, CORT, UA, IL-6, IL-1β, and serum FITC-d levels to assess the effects of SBPM supplementation on oxidative status, catabolic metabolism, and intestinal permeability in HS-treated broiler chickens. As seen in Table 3, all the parameters were increased by chronic HS (#Temp. *p* < 0.001). The SBPM supplementation alleviated the HS-induced plasma TBARS values in a partially dose-dependent manner (*p* < 0.05). The SBPM supplementation significantly attenuated the HS-induced plasma CORT and UA concentrations. The SBPM supplementation significantly reduced the IL-6 concentrations in a dose-dependent manner, while the reducing effect was not observed in IL-1β under the HS conditions. The serum FITC-d detection levels after the oral gavage were significantly reduced by the SBPM supplementation under HS conditions.

### 3.3. Meat Quality

Drip loss, glutathione content, and TBARS values were measured as meat quality parameters (Table 4). The percentages of drip loss in breast and thigh muscles were increased by the HS treatment (#Temp., *p* < 0.001), and the SBPM supplementation suppressed the loss in both muscles (#Diet, *p* = 0.003), with the values reaching near normal. The HS treatment reduced the glutathione content in both the breast and thigh muscles (#Temp., *p* < 0.001), and the SBPM supplementation improved the content in the breast muscle tissue. In the thigh muscle tissue, the SBPM effects were not observed (#Diet = 0.0244); however, increasing effects on the glutathione content were observed (*p* < 0.05). The increasing effects of the SBPM supplementation were of a partially dose-dependent manner. Chronic HS treatment increased the TBARS values in both muscle types (#Temp., *p* < 0.001). The SBPM supplementation suppressed the values in a partially dose-dependent manner (#Diet, *p* < 0.001), with the degrees of the suppressing effects more significant in both muscle types of the HS-treated groups (#Temp.×Diet, *p* < 0.001; *p* = 0.0013).

## 4. Discussion

The present study demonstrated that the SBPM supplementation improved the growth performance and meat quality parameters of the HS-treated birds, with most parameters indicating a dose-dependent manner. There was little information regarding the effects of the SBPM on the harmful effects of HS in chickens; however, one study has reported the beneficial effects of SBPM supplementation on the growth performance, meat quality, and blood-gas parameters of HS-treated chickens [18]. The results obtained in the present study were in agreement with those of the previous investigation, although the supplemental dosage was higher in the previous study (2–10 g/kg diet) than in the present study (75–300 ppm). This difference may be attributed to the extraction and processing methods that differed between the additives used. The present study was the first to demonstrate the alleviating effects of SBPM supplementation on the aggravated oxidative and inflammatory statuses induced by HS treatment.

The SBPM supplementation did not exhibit a remarkable effect on BW under TN conditions throughout most feeding phases, suggesting that the breeding conditions may have been safe and hygienic in the investigation. The present study found that the BW-increasing effects of the SBPM supplementation were more significant at 11–25 d than in the subsequent phases under the HS conditions. Chickens promote heat-dissipation activities and hormonal changes when the atmospheric temperature rises. It has been shown that hormonal changes (T_3_, T_4_, and CORT) and mitochondrial free radical production have been markedly increased at the initial stage of HS treatment [19,20,21,22]. These findings suggest that marked alterations in physiology and metabolism may occur during acclimation to hyperthermic conditions. Therefore, it could be considered that SBPM supplementation effectively suppresses the harmful effects of HS that occur at the beginning. Our previous investigation using isoquinoline alkaloids showed that the growth-promoting effects were more significant in the later feeding phase under HS conditions [17], and a similar effect was observed in an HS study using another SB-derived product [18]. The differences in time to the efficacy in the investigations could be attributable to the chemical character, the processing method, the compositions, or the purity of the additives. The SBPM used in the present study may exert effects relatively quickly under HS conditions.

Chicken meat quality is generally determined by drip loss (water-holding capacity), color, pH, mechanical character (shear force value), aroma, or sensory parameters (tenderness, juiciness, flavor) [23]. It is also known that meat quality is influenced by dietary lipids, amino acids, and vitamins E and D_3_ in diets [24,25]. Moreover, panting, acid/base balance, oxidative status, and hypersecretion of CORT are factors that affect the meat quality in HS conditions [26]. While the impacts of oxidative damage on meat quality have not been completely clarified, one study has suggested that protein oxidation lowers intramuscular protein solubility and the ability to bind water, resulting in increased drip loss [27]. That study also suggested that CORT induces oxidative damage, which is involved in the incidence of PSE-like meat. These findings allowed us to consider the possible machinery governing the improving effects of the SBPM on HS-induced muscle drip loss. The present study found a positive correlation between muscle oxidative damage and muscle drip loss of breast and thigh muscles in HS-treated chickens (Pearson’s coefficients: breast, *r* = 0.547, *p* < 0.01; thigh, *r* = 0.369, *p* < 0.05), suggesting that muscle oxidative status could have negatively influenced muscle drip loss in this study. It can also be suggested that the reduced CORT secretion or increased muscle glutathione content due to the SBPM supplementation suppressed the muscle oxidative damage in the HS-treated birds. For the latter factor, the present study found a negative correlation between the antioxidative peptide content and TBARS values in the breast and thigh muscles of the HS-treated birds (breast, *r* = −0.542, *p* < 0.01; thigh, *r* = −0.398, *p* < 0.05). This suggests that increased glutathione content with SBPM supplementation could be involved in the improvement of oxidative status of muscle tissues, contributing to an improvement of meat quality in HS-treated chickens. It should also be noted that the increased mechanism of the glutathione was due to the SBPM supplementation. Glutathione is synthesized from glutamate, cysteine, and glycine, whose binding reactions are catalyzed by γ-glutamylcysteine synthase [EC 6.3.2.2] and glutathione synthase [EC 6.3.2.3]. It has been reported that HS has reduced the gene expression levels of glutathione synthase in breast muscles and the levels were upregulated by methionine supplementation [28]. From the findings, it might be suggested that the recovery effects of the SBPM on FI promote glutathione synthesis under HS conditions. Meanwhile, the SBPM that was absorbed into the muscle tissues may have scavenged free radicals instead of glutathione, consequently sparing the use of the tripeptide. Further analysis of several biochemical parameters is required to clarify the precise antioxidative machinery of the SBPM and the effects on drip loss under HS conditions.

The present study found that SBPM supplementation reduced HS-induced CORT hypersecretion. Glucocorticoids are secreted from the adrenal gland, which is controlled by the hypothalamic–pituitary–adrenal (HPA) axis. As several physiological stressors stimulate the secretion in chickens [29], this hormone is often considered a stress hormone. The proteolytic effects of CORT on skeletal muscle tissues are well-known, and UA is an end product of nitrogen metabolism in birds. It has been reported that HS-induced CORT and UA have been suppressed concomitantly by isoquinoline alkaloid supplementation [17], and a similar effect was observed in the present study using the SBPM. HS is known to cause intestinal oxidative damage and intestinal barrier dysfunction, while dietary anti-inflammatory treatment attenuates these detrimental effects [17,30]. These findings allowed us to consider that the anti-inflammatory effects of the SBPM on intestinal tissues could suppress HS-induced intestinal hyperpermeability. Meanwhile, excess glucocorticoid administration induces intestinal permeability [31,32] and cytokines stimulate the HPA axis [33,34]. From these lines of evidence, it could also be suggested that reduced plasma inflammatory cytokine levels and CORT secretion by SBPM supplementation are concomitantly associated with suppressed HS-induced intestinal hyperpermeability.

Inflammation and oxidative stress are closely related and are involved in enteric disease in broiler chickens and young pigs [35]. Polyphenols have potent antioxidant power and are widely used to promote growth performance and reduce oxidative stress and inflammation in several stress conditions [6]. The SB-derived polyphenol mixture also has potent antioxidant power, measured as oxygen radical absorbance capacity [18]. These findings allowed us to consider that the beneficial effects of the SBPM on the HS-treated birds could have been due to its potent antioxidant. However, it is well-known that the concentration of phytochemicals and their metabolites in the blood and tissues is very low; only 2% to 15% of their compounds can be absorbed in the small intestine [36,37]. However, phytochemicals exerting potent antioxidant power are known to improve oxidative status in the body. Therefore, it is reasonable to assume that the beneficial effects of the SBPM could be attributed to its involvement in intestinal integrity and health. It has been reported that SB extract has improved intestinal villus and enterocyte structure in chickens [38]. Thus, it could be suggested that improved intestinal permeability by SBPM supplementation prevents the incorporation of pathogens and pathogen-derived components into the circulation, reducing plasma and muscle oxidative damage and circulating inflammatory cytokine concentration. These systemic effects were suggested by our previous investigation [17].

The present study was a preliminary trial and did not, therefore, aim to clarify the underlying mechanism governing the beneficial effects of SBPM supplementation on inflammation and oxidative damage. It has been reported that cultivating chicken-derived polymorphonuclear cells with sugarcane extract has increased their phagocytosis, and orally administrated chickens have exhibited increased antibody responses [39]. One might consider that the (poly)phenolic compounds and polysaccharides of sugarcane exhibit prebiotic effects on the intestinal microbiota [40,41]. Further investigations into the bioavailability and chemical structure/character of the functional components of the SBPM, intestinal microbiota, and intestinal morphology are needed.

## 5. Conclusions

This study demonstrated that SBPM supplementation can improve the growth performance, meat quality, inflammation, and intestinal permeability of chronic HS-treated broiler chickens. Further investigation into the mode of action of the SBPM is needed.

## Figures and Tables

**Table 1:** Diet compositions (%).

Ingredient (Bird Age/Stage)	0–10 d (Starter)	11–25 d (Grower)	26–35 d (Finisher-1)	36–42 d (Finisher-2)
Corn	49.410	51.410	55.990	58.490
Sorghum	10.000	10.000	10.000	10.000
Soybean meal	26.500	27.500	23.500	21.000
Corn gluten meal (CP60)	5.000	3.000	3.000	3.000
Fish meal (CP65)	4.000	3.000	3.000	3.000
Vegetable oil	1.000	1.900	1.900	2.000
Salt	0.330	0.350	0.340	0.350
CaHPO_4_	1.500	1.050	0.800	0.700
Calcium bicarbonate	0.980	0.750	0.650	0.600
L-Lysine hydrochloride	0.300	0.220	0.180	0.180
DL-Methionine	0.330	0.320	0.210	0.250
L-Threonine	0.140	0.100	0.070	0.070
Choline chloride	0.080	0.060	0.060	0.060
Selenium	0.030	0.030	0.030	0.030
Vitamin/mineral mix *	0.400	0.310	0.270	0.270
**Calculated values**				
Crude protein (%)	23.0	21.5	20.0	19.0
Metabolizable energy (kcal/kg)	3000	3050	3100	3200
Calcium (%)	0.99	0.76	0.66	0.61
Nonphytate phosphorus (%)	0.52	0.43	0.37	0.35
Lysine (%)	1.33	1.21	1.10	1.04
Methionine/cysteine (%)	1.01	0.94	0.82	0.83
Threonine (%)	0.88	0.80	0.72	0.70

* Components are as follows: vitamin A, 11,000 IU; vitamin D3, 4500 IU; DL-α-tocopherol acetate, 65 mg; 2-methyl-1,4-naphthoquinone sodium bisulfite, 6.91 mg; thiamin nitrate, 4.94 mg; riboflavin, 8.0 mg; pyridoxine hydrochloride, 4.86 mg; nicotinamide, 64.5 mg; D-calcium pantothenate, 21.7 mg; folic acid, 2.0 mg; cyanocobalamin, 18 μg; D-biotin, 0.28 mg; manganese(II) sulfate, 330 mg; anhydrous iron(II) sulfate, 54.4 mg; anhydrous copper sulfate, 40.2 mg; zinc carbonate, 120 mg; calcium iodate, 1.92 mg.

**Table 2:** Growth performances of thermoneutral and heat-stressed broiler chickens fed different levels of SBPM.

Age (Period)	Thermoneutral, SBPM (ppm)				Heat-Stressed, SBPM (ppm)				SEM	ANOVA		
	0	75	150	300	0	75	150	300		Temp.	Diet	Temp. × Diet
Body weight, g												
10 d	336	336	328	337	-	-	-	-	4	-	0.107	-
25 d	1415 ^a^	1425 ^a^	1424 ^a^	1409 ^a^	1179 ^c^	1157 ^c^	1224 ^b^	1383 ^a^	24	*p* < 0.001	*p* < 0.001	*p* < 0.001
35 d	2313 ^b^	2437 ^a^	2475 ^a^	2409 ^ab^	1998 ^d^	1988 ^d^	2110 ^c^	2294 ^b^	37	*p* < 0.001	*p* < 0.001	*p* < 0.001
42 d	3168 ^a^	3255 ^a^	3300 ^a^	3233 ^a^	2686 ^d^	2710 ^cd^	2849 ^c^	3016 ^b^	54	*p* < 0.001	*p* < 0.001	*p* < 0.001
Body weight gain, g												
0–10 d	292	293	285	294	-	-	-	-	4	-	0.103	-
11–25 d	1080 ^a^	1085 ^a^	1097 ^a^	1076 ^a^	843 ^c^	825 ^c^	894 ^b^	1043 ^a^	25	*p* < 0.001	*p* < 0.001	*p* < 0.001
26–35 d	898	1011	1051	1000	819	832	886	911	45	*p* < 0.001	*p* < 0.001	0.283
36–42 d	855	818	825	824	688	722	739	722	61	*p* < 0.001	0.993	0.7792
0–42 d	3125 ^a^	3212 ^a^	3257 ^a^	3190 ^a^	2643 ^d^	2667 ^c^	2806 ^c^	2973 ^b^	54	*p* < 0.001	*p* < 0.001	*p* < 0.001
Feed intake, g												
0–10 d	297	297	293	301	-	-	-	-	7	-	0.620	-
11–25 d	1402 ^a^	1394 ^a^	1380 ^a^	1384 ^a^	1222 ^b^	1219 ^b^	1235 ^b^	1372 ^a^	40	*p* < 0.001	0.038	0.015
26–35 d	1461 ^b^	1535 ^a^	1534 ^a^	1531 ^a^	1384 ^c^	1336 ^bc^	1411 ^b^	1439 ^b^	33	*p* < 0.001	0.032	0.048
36–42 d	1539	1445	1460	1500	1366	1322	1325	1311	42	*p* < 0.001	0.096	0.647
0–42 d	4701	4674	4665	4717	4267	4170	4264	4422	65	*p* < 0.001	0.022	0.173
Feed conversion ratio												
0–10 d	1.02	1.01	1.03	1.03	-	-	-	-	0.02	-	0.937	-
11–25 d	1.30	1.29	1.26	1.29	1.45	1.48	1.38	1.32	0.05	*p* < 0.001	0.067	0.126
26–35 d	1.63	1.52	1.47	1.54	1.71	1.61	1.60	1.58	0.06	0.012	0.022	0.797
36–42 d	1.86	1.81	1.78	1.83	2.02	1.85	1.83	1.83	0.16	0.463	0.659	0.918
0–42 d	1.51 ^b^	1.46 ^bc^	1.43 ^c^	1.48 ^b^	1.62 ^a^	1.57 ^ab^	1.52 ^b^	1.49 ^b^	0.03	*p* < 0.001	0.003	0.047

Data are means of six replicates (6 birds per replicate). Data were analyzed by one-way (stater) or two-way (grower, finisher-1/-2, total) ANOVA with Tukey’s multiple-comparison test. Different superscript letters indicate statistically significant differences (^abcd^ *p* < 0.05).

**Table 3:** Blood biochemical parameters of broiler chickens fed different levels of SBPM.

Parameters	Thermoneutral (SBPM, ppm)				Heat-Stressed (SBPM, ppm)				SEM	Two-Way ANOVA		
	0	75	150	300	0	75	150	300		Temp.	Diet	Temp. × Diet
TBARS	38.6 ^c^	31.7 ^d^	31.1 ^d^	33.8 ^cd^	58.2 ^a^	50.7 ^b^	48.1 ^bc^	43.2 ^c^	2.7	*p* < 0.001	*p* < 0.001	0.045
CORT	24.3 ^c^	26.2 ^c^	25.1 ^c^	28.1 ^c^	53.8 ^a^	51.4 ^a^	48.3 ^ab^	40.6 ^b^	2.8	*p* < 0.001	0.019	0.047
UA	77.6 ^c^	80.1 ^c^	82.2 ^bc^	76.4 ^c^	101.5 ^a^	86.7 ^b^	88.0 ^b^	85.8 ^b^	3.5	*p* < 0.001	0.012	0.002
IL-6	37.0 ^d^	35.7 ^d^	34.0 ^d^	36.7 ^d^	87.6 ^a^	73.4 ^b^	66.4 ^bc^	62.4 ^c^	3.0	*p* < 0.001	*p* < 0.001	*p* < 0.001
IL-1β	9.5	8.2	8.0	8.3	29.7	25.9	22.0	20.7	2.5	*p* < 0.001	0.024	0.135
FITC-d	0.28 ^c^	0.32 ^c^	0.32 ^c^	0.31 ^c^	0.64 ^a^	0.48 ^b^	0.52 ^b^	0.50 ^b^	0.03	*p* < 0.001	0.023	*p* < 0.001

Data are means of twelve birds. Data were analyzed using two-way ANOVA with Tukey’s multiple-comparison test. Different superscript letters indicate statistically significant differences (^abcd^ *p* < 0.05). A unit of each parameter is as follows: TBARS, nmol/mL; FITC-d, ug/mL; CORT, ng/mL; UA, nmol/mL; IL-6, pg/mL; IL-1β, pg/mL. Abbreviations: TBARS, 2-thiobarbituric acid reactive substance; FITC-d, fluorescein isothiocyanate-dextran; CORT, corticosterone; UA, uric acid; IL, interleukin.

**Table 4:** Effects of different levels of SBPM supplementation on meat quality of broiler chickens.

Parameters	Thermoneutral (SBPM, ppm)				Heat-Stressed (SBPM, ppm)				SEM	Two-Way ANOVA		
	0	75	150	300	0	75	150	300		Temp.	Diet	Temp. × diet
Drip loss, %												
Breast	1.92	1.63	1.44	1.48	4.07	3.75	2.66	3.26	0.3	*p* < 0.001	0.003	0.217
Thigh	1.96	1.71	1.89	2.03	3.74	3.51	2.94	3.05	0.3	*p* < 0.001	0.336	0.197
Glutathione, μmol/g wet tissue												
Breast	2.70 ^a^	2.65 ^ab^	2.69 ^a^	2.70 ^a^	2.12 ^c^	2.30 ^c^	2.43 ^cb^	2.46 ^b^	0.1	*p* < 0.001	0.0365	0.0347
Thigh	4.01 ^a^	3.88 ^a^	4.05 ^a^	3.85 ^a^	2.57 ^bc^	2.61 ^b^	2.78 ^ab^	2.97 ^a^	0.1	*p* < 0.001	0.2044	0.0432
TBARS, nmol/g wet tissue												
Breast	28.2 ^c^	23.5 ^cd^	21.1 ^d^	24.9 ^cd^	66.3 ^a^	55.4 ^b^	43.6 ^c^	37.1 ^c^	2.7	*p* < 0.001	*p* < 0.001	*p* < 0.001
Thigh	78.6 ^c^	69.3 ^cd^	60.3 ^d^	68.0 ^cd^	110.0 ^a^	95.4 ^b^	87.5 ^c^	83.3 ^c^	2.7	*p* < 0.001	*p* < 0.001	0.0013

Data are means of twelve birds. Data were analyzed using two-way ANOVA with Tukey’s multiple-comparison test. Different superscript letters indicate statistically significant differences (^abcd^ *p* < 0.05).

## Data Availability

The raw data supporting the conclusions of this article will be made available by the authors on request.
